# Implications of iron deficiency/anemia on the classification of diabetes using HbA1c

**DOI:** 10.1038/nutd.2015.16

**Published:** 2015-06-22

**Authors:** S M Attard, A H Herring, H Wang, A-G Howard, A L Thompson, L S Adair, E J Mayer-Davis, P Gordon-Larsen

**Affiliations:** 1 Department of Nutrition, Gillings School of Global Public Health, UNC-Chapel Hill, Chapel Hill, NC, USA; 2 Carolina Population Center, UNC-Chapel Hill, Chapel Hill, NC, USA; 3 Department of Biostatistics, Gillings School of Global Public Health, UNC-Chapel Hill, Chapel Hill, NC, USA; 4 National Institute of Nutrition and Food Safety, Chinese Center for Disease Control and Prevention, Beijing, China; 5 Department of Anthropology, UNC-Chapel Hill, Chapel Hill, NC, USA; 6 Department of Medicine, UNC-Chapel Hill, Chapel Hill, NC, USA

## Abstract

**Background/Objectives:**

Nonglycemic factors like iron deficiency (ID) or anemia may interfere with classification of diabetes and prediabetes using hemoglobin A1c (HbA1c). However, few population-based studies of diabetes in areas with endemic ID/anemia have been conducted. We aimed to determine how mutually exclusive categories of ID alone, anemia alone and iron-deficiency anemia (IDA) were each associated with prediabetes and diabetes prevalence using fasting blood glucose (FBG) versus HbA1c in a population-based study of adults with endemic ID/anemia.

**Subjects/Methods:**

We used data from the China Health and Nutrition Survey, a longitudinal, population-based study across 228 communities within nine provinces of China. This analysis included 7308 adults seen in the 2009 survey aged 18–75 years. We used descriptive and covariate-adjusted models to examine relative risk of prediabetes and diabetes using FBG alone, HbA1c alone, HbA1c and FBG, or neither (normoglycemia) by anemia alone, ID alone, IDA or normal iron/hemoglobin.

**Results:**

Approximately 65% of individuals with diabetes in our sample were concordantly classified with diabetes using both FBG and HbA1c, while 35% had a discordant diabetes classification: they were classified using either FBG or HbA1c, but not both. Fewer participants with ID alone versus normal iron/hemoglobin were classified with diabetes using HbA1c only. From covariate-adjusted, multinomial regression analyses, the adjusted prevalence of prediabetes using HbA1c only was 22% for men with anemia alone, but 13% for men with normal iron/hemoglobin. In contrast, the predicted prevalence of prediabetes using HbA1c only was 8% for women with ID alone, compared with 13% for women with normal iron/hemoglobin.

**Conclusions:**

These findings suggest potential misclassification of diabetes using HbA1c in areas of endemic ID/anemia. Estimating diabetes prevalence using HbA1c may result in under-diagnosis in women with ID and over-diagnosis in men with anemia.

## Introduction

Fasting blood glucose (FBG) and hemoglobin A1c (HbA1c) are important diagnostic measures to assess glycemia; however, HbA1c is increasingly recommended for use in population-based settings because it does not require fasting and has low intra-individual variability.^1^ In the general population, HbA1c and FBG do not classify diabetes identically because they reflect glycemia status over different time periods.^2, 3^ It is well established that HbA1c levels can be affected by conditions unrelated to diabetes including anemia, blood loss and iron deficiency (ID).^1^ Anemia affects an estimated five million women of reproductive age in the US^4^ and 1.6 billion individuals worldwide,^4^ a substantial portion of whom have concurrent ID.^5^ Thus, there is potential for diabetes misclassification across many populations worldwide. To understand the clinical implications of ID and/or anemia for diabetes prevalence estimates when using HbA1c, population-based research in areas with a high prevalence of ID and/or anemia is needed.

The magnitude and direction of diabetes misclassification due to ID and anemia is not well understood. The mechanism through which ID and anemia influences HbA1c has yet to be fully elucidated;^5, 6, 7, 8^ however, most epidemiologic studies suggest that iron-deficiency anemia (IDA) can result in spuriously high HbA1c values,^6, 7, 9, 10^ though some suggest there is lower HbA1c among individuals with IDA^11^ or anemia.^12^ These differences may relate to the multiple etiologies for anemia, which include ID, sickle cell disease or other thalassemias, vitamin B12 deficiency or folate deficiency.^5^ ID may be caused by insufficient iron intake, inability to absorb iron, blood loss, menstrual blood loss or pregnancy.^5^ With these multiple etiologies, IDA may represent two separate disease processes, anemia and ID. Furthermore, susceptibility to, or severity of, ID versus anemia may differ by sex because of menstruation in women. Few population-based studies have separated ID and anemia into mutually exclusive categories to examine how each may differentially impact prediabetes and diabetes prevalence estimates using HbA1c.

Thus, we examined how anemia alone, ID alone and IDA were each associated with the prevalence of prediabetes and diabetes when using FBG and HbA1c in a population-based sample of 7308 Chinese adults aged 18–75 years from the China Health and Nutrition Survey. We examined the variation in the prevalence of prediabetes or diabetes using descriptive and covariate-controlled analyses, hypothesizing that IDA would result in over-diagnosis of diabetes using HbA1c versus FBG.

## Materials and methods

### Study design and participants

The China Health and Nutrition Survey is a longitudinal study across 228 communities within nine provinces of China. Surveys began in 1989, with subsequent surveys every 2–4 years, for a total of nine rounds between 1989 and 2011. The China Health and Nutrition Survey was designed to provide representation of rural, urban and suburban areas varying substantially in geography, economic development, public resources and health indicators,^13^ and it is the only large-scale, longitudinal study of its kind in China. The original survey in 1989 used a multistage, random cluster design in eight provinces (Liaoning, Jiangsu, Shandong, Henan, Hubei, Hunan, Guangxi and Guizhou) to select a stratified probability sample; a ninth province, Heilongjiang, was added in 1997 using a similar sampling strategy. Essentially, two cities (one large and one small city—usually the provincial capital and a lower income city) and four counties (stratified by income: one high, one low and two middle income counties) were selected in each province. Within cities, two urban and two suburban communities were selected; within counties, one community in the capital city and three rural villages were chosen. Twenty households per community were then selected for participation. The study met the standards for the ethical treatment of participants and was approved by the Institutional Review Boards of the University of North Carolina at Chapel Hill and the Institute of Nutrition and Food Safety, Chinese Center for Disease Control and Prevention. More detailed survey procedures can be found elsewhere.^13^

Adults aged 18–75 years with a blood draw at the 2009 China Health and Nutrition Survey exam were eligible for inclusion (*n*=8102). Of these 8102 adults, individuals were excluded due to: pregnancy at the time of survey (*n*=62), having a nonfasting blood measurement (*n*=354) or missing data on one or more measures: HbA1c (*n*=56), FBG (*n*=19), hemoglobin (*n*=32), ferritin (*n*=11), transferrin receptor (*n*=11), C-reactive protein (*n*=1), waist circumference (*n*=222), household income (*n*=101) or smoking status (men only; *n*=30), with some individuals missing information for more than one variable (total *n*=378), leaving 7308 individuals in our analytic sample. Of the full eligible sample (*n*=8102), a greater proportion of those included (versus excluded) in the analytic sample (*n*=7308) were younger, had lower FBG and were from lower urbanicity areas.

### Measures

After an overnight fast, blood was collected by venipuncture (12 ml). Whole blood was immediately centrifuged and serum tested for glucose using a Hitachi 7600 analyzer using a glucose oxidase phenol 4-aminoantipyrine peroxidase kit (GOD-PAP; Randox, Crumlin, UK). HbA1c was measured at a central lab in each province with a high-performance liquid chromatography system (D10 HLC, Bio-Rad, Hercules, CA, USA; PDQ HPLC, Primus, Kansas City, MO, USA; Model HLC-723 G7, Tosoh Corporation, Tokyo, Japan). All the samples were calibrated to the D10 HLC from Bio-Rad. Self-report questionnaires were used to ascertain medical history and current medication use. We compared prevalent diabetes using HbA1c (HbA1c ⩾6.5% (48 mmol mol^−1^)) versus FBG (FBG ⩾126 mg dl^−1^ (7.0 mmol l^−1^)).^1^ We considered individuals reporting diabetes diagnosis (answering ‘yes' to the question ‘has a doctor ever told you that you suffer from diabetes?') and treatment (reporting diabetes medication use) as having diabetes. For prevalent prediabetes, we used HbA1c (HbA1c=5.7–6.5% (39–48 mmol mol^−1^)) versus FBG (FBG=100–126 mg dl^−1^ (5.5–7.0 mmol l^−1^)) according to the American Diabetes Association guidelines,^1^ excluding individuals classified with diabetes according to any criteria (HbA1c, FBG, diabetes diagnosis or diabetes medication). In descriptive analyses and multinomial logistic regression models, we categorized individuals according to their diabetes status (nondiabetic, diabetes_FBG_ (discordant, by FBG only), diabetes_HbA1c_ (discordant, by HbA1c only) or diabetes_HbA1c+FBG_ (concordant by FBG and HbA1c)) and prediabetes status (normoglycemia, prediabetes_FBG_, prediabetes_HbA1c_ or prediabetes_HbA1c+FBG_).

Hemoglobin and iron status markers were measured at a national central lab in Beijing (medical laboratory accreditation certificate ISO 15 189:2007) with strict quality control. From serum, soluble transferrin receptor was measured via nephelometry on a Siemens BNP (Seimans China, Beijing, China) and ferritin was measured via radioimmunoassay on a Gamma counter XH-6020 (North Institute of Bio-Tech, Beijing, China). From whole blood, hemoglobin was measured via VCS on a Beckman Coulter LH750 (Beckman, Brea, CA, USA). Because acute infection artificially raises ferritin, we followed WHO recommendations and multiplied the measured ferritin values in 802 individuals with a C-reactive protein ⩾5 mg l^−1^ by 0.65.^14^ Anemia alone was defined as hemoglobin <12 g dl^−1^ (women) or <13 g dl^−1^ (men) according to WHO guidelines^5^ without ID (defined as inflammation-adjusted ferritin <15 μg l^−1^ or transferrin receptor ⩾1.76 mg l^−1^ without anemia).^5^ IDA was defined as having anemia and ID according to WHO recommendations.^15^

Age, sex, smoking status and pregnancy status/history were self-reported at each survey. Household income was derived from individual and household questionnaires from time-use, asset and economic activity at each survey and inflated to 2009 Yuan currency in analysis for comparability over time, and categorized into tertiles. Waist circumference was measured midway between the lowest rib and the iliac crest using nonelastic tape. Urbanicity, which reflects the degree of modernization of each of the China Health and Nutrition Survey communities, was measured using a multicomponent scale incorporating infrastructure, economic and social service domains (range: 0–120). The scale has high reliability and validity,^16^ with higher urbanicity score corresponding to having more ‘urban' characteristics across multiple domains. We categorized urbanicity into tertiles. We controlled for region (North, Central, South) due to regional differences in geography and diet across China.

### Statistical analyses

#### Descriptive analyses

All analyses were conducted in Stata 13 (Stata Corp, College Station, TX, USA). First, we examined the differences in sample characteristics by sex, using *χ*^2^ or analysis of variance tests for categorical or continuous variables, respectively. Second, we examined sex-specific differences in diabetes classification (nondiabetic, diabetes_HbA1c_, diabetes_FBG_ or diabetes_HbA1c+FBG_) or prediabetes classification (normoglycemia, prediabetes_HbA1c_, prediabetes_FBG_ or prediabetes_HbA1c+FBG_) according to ID and/or anemia status (normal iron/hemoglobin, anemia alone, ID or IDA), testing group-level differences in the classification via *χ*^2^ tests. Third, among individuals with diabetes or prediabetes, we examined the mismatch between the prevalence of diabetes and prediabetes (separately) on the basis of HbA1c versus FBG (concordant by HbA1c and FBG; discordant by FBG only; discordant by HbA1c only) using pairwise *χ*^2^ tests comparing categories of ID alone, anemia alone or IDA with normal iron/hemoglobin as referent. Statistical significance was set at the *P*<0.05 level for all descriptive analyses.

#### Statistical modeling

We used sex-specific multinomial logistic regression models to examine the estimated relative risk ratios (RRR) of diabetes using concordant and discordant classification by HbA1c and FBG (diabetes_FBG_, diabetes_HbA1c_, diabetes_HbA1c+FBG_ and nondiabetic (referent)) across categories of ID and/or anemia status, controlling for confounding by demographic, social and environmental factors. An identical set of models predicted relative risk of concordant and discordant prediabetes classification using HbA1c and FBG (prediabetes_FBG_, prediabetes_HbA1c_, prediabetes_HbA1c+FBG_ and normoglycemia (referent)), excluding *n*=737 individuals with diabetes according to either FBG, HbA1c, doctor diagnosis or diabetes medication use. We stratified models by sex because cutpoints for anemia and ID differ by sex and because interaction terms between categories of ID/anemia status and sex were statistically significant (*P*<0.05). Final models included age (linear), urbanicity (low, medium, high), number of cigarettes smoked per day (linear and quadratic; men only due to low smoking prevalence (3%) in women), pregnancy history (ever versus never; women only), household income (low, medium, high), region (North, Central, South) and waist circumference (linear). Models were clustered at the community level using a Huber–White type variance estimator to correct standard errors.^17^ In figures, we present relative risk ratios (RRRs) from these models and model-based predicted prevalence of diabetes or prediabetes using FBG only, HbA1c only or both FBG and HbA1c across ID/anemia categories.

#### Sensitivity analysis

Because individuals with the genetic hemoglobin E (HbE) variant could have an artificially lower HbA1c relative to individuals without HbE,^18^ we conducted an additional analysis excluding participants from the South region (where the HbE trait is most common) to determine whether relationships among ID, anemia, IDA and diabetes classification by HbA1c versus FBG were different in the presence of the HbE genetic variant.

Because provinces of China vary greatly in climate, altitude, and terrain, potentially affecting biochemical parameters related to hemoglobin and anemia,^19^ we conducted two sensitivity analyses: first, we repeated our analysis stratified by region to see whether the magnitude or direction of association differed by region. Second, we redefined our region variable according to the four regions identified in a paper by Miao, *et al.*^19^ to examine whether our results were sensitive to the classification of region.

### Role of the funding source

The sponsors of the study had no role in study design, data collection, data analysis, data interpretation, writing of the report and the decision to submit for publication. The corresponding author had full access to all the data in the study and had final responsibility for the decision to submit for publication.

## Results

### Prevalence of prediabetes, diabetes, ID and anemia

Approximately 35% of men and women had HbA1c or FBG above the prediabetes thresholds. Although 11.1% of men and 9.2% of women were classified as having diabetes by either FBG, HbA1c, physician diagnosis or medication use, only 6.2% of men and 4.8% of women had diabetes using both HbA1c and FBG, physician diagnosis or medication use (Table 1). The prevalence of ID and/or anemia was ~20% for men and 40% for women.

### Differences in prevalence of diabetes by ID and/or anemia status

In unadjusted analyses, there was a statistically significant difference in diabetes (men: *P*=0.04; women: *P*=0.008) and prediabetes (men: *P*<0.001; women: *P*<0.001) classification using FBG versus HbA1c across categories of ID and/or anemia status (Table 2).

**Figure 1:**
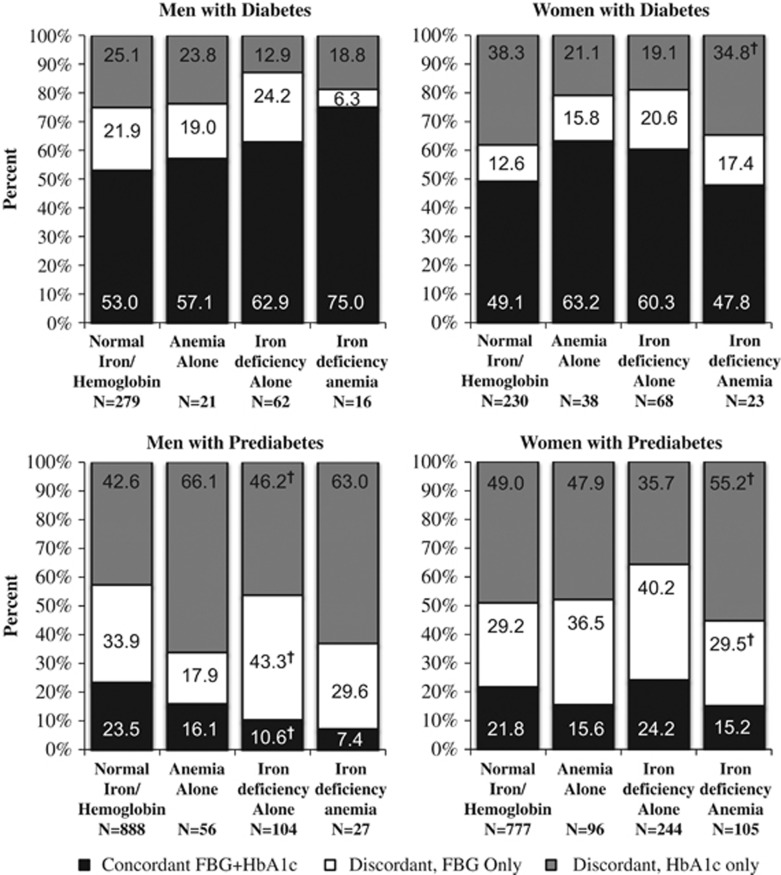
Discordance and concordance in classification of diabetes and prediabetes using HbA1c only, FBG only or with both HbA1c and FBG by iron/anemia status in Chinese adults aged 18–75 years from the 2009 China Health and Nutrition Survey with diabetes or prediabetes. Diabetes classified as concordant HbA1c+FBG (FBG ⩾126 mg dl^−1^ and HbA1c ⩾6.5% (48 mmol mol^−1^) or doctor diagnosis), discordant HbA1c alone (HbA1c ⩾6.5% and FBG <126 mg dl^−1^ (48 mmol mol^−1^) with no doctor diagnosis) or discordant FBG alone (FBG ⩾126 mg dl^−1^ and HbA1c <6.5% (48 mmol mol^−1^) with no doctor diagnosis). Prediabetes classified as concordant HbA1c+FBG (FBG=100–126 mg dl^−1^ and HbA1c=5.7–6.5% (39–48 mmol mol^−1^)), discordant HbA1c alone (HbA1c=5.7–6.5% (39–48 mmol mol^−1^) and FBG <100 mg dl^−1^) or discordant FBG alone (FBG=100–126 mg dl^−1^ and HbA1c <5.7% (39 mmol mol^−1^)). Iron/anemia status was categorized as normal iron and hemoglobin (hemoglobin ⩾12 g dl^−1^ (women) or ⩾13 g dl^−1^ (men), ferritin ⩾15 μg l^−1^, soluble transferrin receptor <1.76 mg l^−1^), anemia alone (as hemoglobin <12 g dl^−1^ (women) or <13 g dl^−1^ (men) without iron deficiency), iron deficiency alone (ferritin <15 μg l^−1^ or transferrin receptor ⩾1.76 mg l^−1^ without anemia) and iron-deficiency anemia (anemia: hemoglobin <12 g dl^−1^ (women) or <13 g dl^−1^ (men), as well as iron-deficiency ferritin <15 μg l^−1^ or transferrin receptor ⩾1.76 mg l^−1^). ^†^*P*<0.05, denotes statistically significant difference relative to normal iron/hemoglobin in the percent of individuals classified as concordant FBG and HbA1c, discordant HbA1c only or discordant FBG only using a chi-squared test. Percents may not add up to 100% due to rounding.

Among individuals with diabetes, fewer individuals with ID alone (men: 12.9% women: 19.1%) were estimated to have diabetes using HbA1c only (diabetes_HbA1c_) compared with individuals with normal iron/hemoglobin (men: 25.1% women: 38.3% *P*<0.05; Figure 1). However, among individuals with prediabetes, more men with anemia alone (66.1%) versus normal iron/hemoglobin (42.2% *P*<0.05) were estimated to have prediabetes_HbA1c_. Among individuals with prediabetes, more individuals with IDA (men: 63% women: 55.2%) versus normal iron/hemoglobin (men: 42.6% women: 49.0%) were estimated to have prediabetes_HbA1c_.

**Figure 2:**
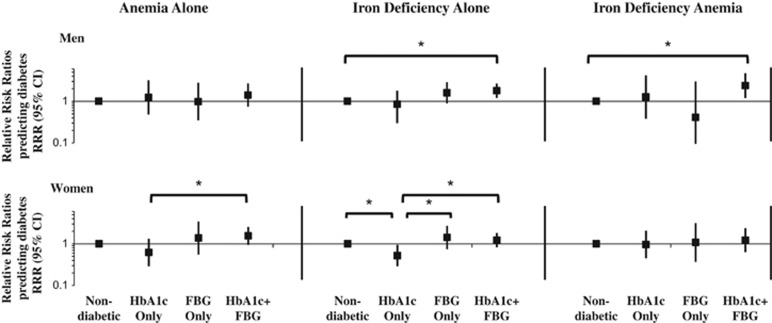
Multinomial logistic regression models predicting discordance and concordance in classification of diabetes using HbA1c only, FBG only, or both HbA1c and FBG relative to nondiabetic according to iron deficiency and/or anemia status, from the 2009 China Health and Nutrition Survey. Diabetes outcome was classified as nondiabetic (FBG <126 mg dl^−1^ and HbA1c <6.5% (48 mmol mol^−1^)), HbA1c only (HbA1c ⩾6.5% (48 mmol mol^−1^) and FBG <126 mg dl^−1^), FBG only (FBG ⩾126 mg dl^−1^ and HbA1c <6.5% (48 mmol mol^−1^)) or HbA1c+FBG (FBG ⩾126 mg dl^−1^ and HbA1c ⩾6.5% (48 mmol mol^−1^), doctor diagnosis of diabetes or reported diabetes medication use). Main exposure variable was categories of iron deficiency/anemia status. Models were clustered at the community level and adjusted for age, urbanicity level (low, medium, high), number of cigarettes smoked per day and number of cigarettes squared (men only), ever pregnant (ever versus never; women only), household income (low, medium, high), region (North, Central, South) and waist circumference. *P<0.05, denotes statistically significant difference in the relative risk ratios using chi-squared tests. FBG, fasting blood glucose; IDA, iron-deficiency anemia; RRR, relative risk ratio.

**Figure 3:**
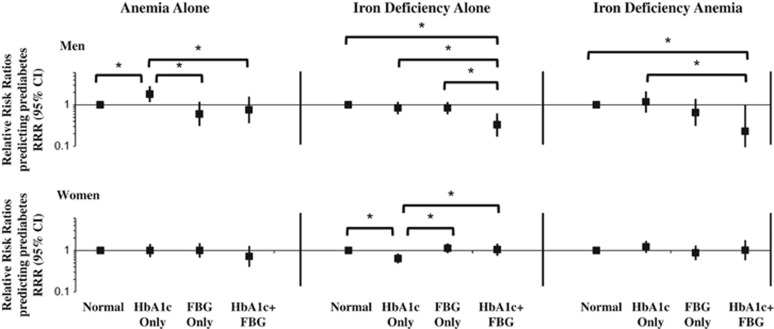
Multinomial logistic regression models predicting discordance and concordance in classification of prediabetes using HbA1c only, FBG only or both HbA1c and FBG relative to normoglycemia according to iron deficiency and/or anemia status, from the 2009 China Health and Nutrition Survey. Prediabetes model excluded *N*=737 individuals with diabetes according to FBG, HbA1c, doctor diagnosis or reported diabetes medication use. Prediabetes outcome was classified as normal (FBG <100 mg dl^−1^ and HbA1c <5.7% (39 mmol mol^−1^)), HbA1c only (HbA1c=5.7–6.5% (39–48 mmol mol^−1^) and FBG <100 mg dl^−1^), FBG only (FBG=100–126 mg dl^−1^ and HbA1c <5.7% (39 mmol mol^−1^)) or HbA1c+FBG (FBG=100–126 mg dl^−1^ and HbA1c =5.7–6.5% (39–48 mmol mol^−1^)). Main exposure variable was categories of iron deficiency/anemia status. Models were clustered at the community level and adjusted for age, urbanicity level (low, medium, high), number of cigarettes smoked per day and number of cigarettes squared (men only), ever pregnant (ever versus never; women only), household income (low, medium, high), region (North, Central, South) and waist circumference. *P<0.05, denotes statistically significant difference in the relative risk ratios using chi-squared tests. FBG, fasting blood glucose; IDA, iron-deficiency anemia; RRR, relative risk ratio.

**Figure 4:**
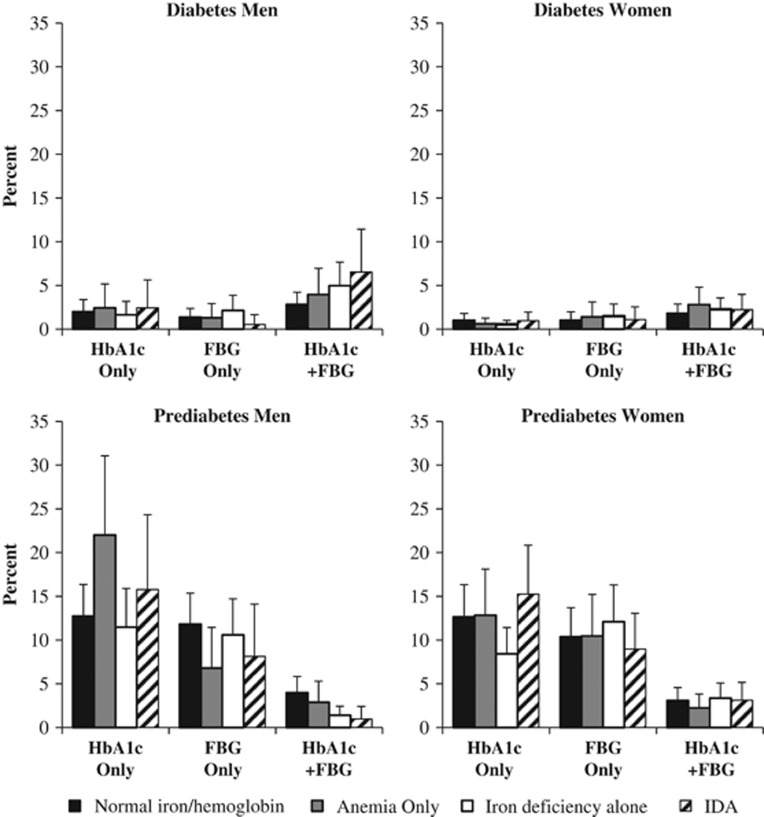
Multinomial logistic regression model-predicted percent of individuals classified with diabetes or prediabetes using HbA1c only, FBG only or both HbA1c and FBG according to iron deficiency and/or anemia status, from the 2009 China Health and Nutrition Survey. Diabetes outcome was classified as nondiabetic (FBG <126 mg dl^−1^ and HbA1c <6.5% (48 mmol mol^−1^)), HbA1c only (HbA1c ⩾6.5% (48 mmol mol^−1^) and FBG <126 mg dl^−1^), FBG only (FBG ⩾126 mg dl^−1^ and HbA1c <6.5% (48 mmol mol^−1^)) or HbA1c+FBG (FBG ⩾126 mg dl^−1^ and HbA1c ⩾6.5% (48 mmol mol^−1^), doctor diagnosis of diabetes or reported diabetes medication use). Prediabetes model excluded *N*=737 individuals with diabetes according to FBG, HbA1c, doctor diagnosis or reported diabetes medication use. Prediabetes outcome was classified as normoglycemia (FBG <100 mg dl^−1^ and HbA1c <5.7% (39 mmol mol^−1^)), HbA1c only (HbA1c=5.7–6.5% (39–48 mmol mol^−1^) and FBG <100 mg dl^−1^), FBG only (FBG=100–126 mg dl^−1^ and HbA1c <5.7% (39 mmol mol^−1^)) or HbA1c+FBG (FBG=100–126 mg dl^−1^ and HbA1c =5.7–6.5% (39–48 mmol mol^−1^)). Main exposure variable was categories of iron deficiency/anemia status. Models were clustered at the community level and adjusted for age, urbanicity level (low, medium, high), number of cigarettes smoked per day and number of cigarettes squared (men only), ever pregnant (ever versus never; women only), household income (low, medium, high), region (north, central, south) and waist circumference. Patterns of statistical significance can be found in Figures 2 and 3. ID, iron deficiency; IDA, iron-deficiency anemia.

Using covariate-adjusted models, we examined differences in the classification of diabetes and prediabetes (separately) using HbA1c versus FBG across mutually exclusive categories of ID and/or anemia (Figures 2,3,4, with RRRs presented in Supplementary Tables 1 and 2). In comparison with nondiabetic individuals, women with ID alone versus normal iron/hemoglobin had lower relative risk of being classified with diabetes_HbA1c_ (RRR=0.52; 95% confidence interval (CI): 0.29, 0.95; Figure 2 and Supplementary Table 1). Similarly, in comparison with diabetes_FBG_, women with ID alone versus normal iron/hemoglobin had lower relative risk of being classified with diabetes_HbA1c_ (RRR=0.37; CI: 0.15, 0.88; Figure 2 and Supplementary Table 1). In comparison with nondiabetic individuals, men with IDA relative to normal iron/hemoglobin had higher relative risk of being concordantly classified with diabetes_HbA1c+FBG_ (RRR=2.38; CI: 1.20, 4.72). A similar pattern was seen for ID alone relative to normal iron/hemoglobin (RRR=1.80; CI: 1.21, 2.67; Figure 2 and Supplementary Table 1).

From the prediabetes models, relative to normoglycemia, men with anemia alone versus normal iron/hemoglobin were more likely to be classified with prediabetes_HbA1c_ (RRR=1.81; CI: 1.16, 2.82; Figure 3 and Supplementary Table 2). Similarly, in comparison with prediabetes_FBG_, men with anemia alone had higher relative risk of being classified with prediabetes_HbA1c_ (RRR=3.00; CI: 1.43, 6.30; Figure 3 and Supplementary Table 2). Men with ID alone or IDA had higher relative risk of prediabetes _HbA1c_ than prediabetes_HbA1c+FBG_. Women with ID alone versus normal iron/hemoglobin were more likely to be classified with prediabetes_FBG_ relative to prediabetes_HbA1c_ (RRR=1.76; CI: 1.25, 2.49). There was relatively little difference in prediabetes by HbA1c versus FBG for women with IDA.

Using these same covariate-adjusted models, we calculated the predicted probability of diabetes and prediabetes classification using FBG only (discordant), HbA1c only (discordant) or both HbA1c and FBG (concordant; Figure 4). Among women with ID alone, a smaller percentage were predicted to have diabetes_HbA1c_ (0.5%) compared with diabetes_FBG_ (1.5%). For men, concordant classification of prediabetes (prediabetes_HbA1c+FBG_) was less common in men with IDA (1.0%) versus normal iron/hemoglobin (4.0%). A higher percentage of men with anemia alone were predicted to have prediabetes_HbA1c_ (22.0%) versus prediabetes_FBG_ (6.8%).

#### Sensitivity analyses

In our analysis to examine differences in findings in the presence of the HbE genetic variant, we excluded participants from the South region (*n*=3323) where the HbE variant is most common. We did not see any differences in the direction or magnitude of associations between ID, anemia or IDA (versus normal iron/hemoglobin) with diabetes or prediabetes classification via HbA1c or FBG, although fewer results were statistically significant due to the smaller sample size in this subsample.

In our analysis with stratified models by region (North, Central and South), we did not see differences in the direction or magnitude of associations, however, estimates were imprecise due to small sample size.

In our last sensitivity analysis, we redefined our region variable according to the four regions identified in a paper by Miao, *et al.*^19^ to examine whether our results were sensitive to the classification of region and did not see differences in the direction or magnitude of associations.

## Discussion

ID-related changes in the hemoglobin molecule as well as anemia-induced erythrocyte turnover differences may result in inaccurate HbA1c measurements for estimates of diabetes prevalence. Though HbA1c levels may not accurately reflect glycemia status if individuals have certain anemias, blood loss or ID, the magnitude and direction of the associations between ID and/or anemia with diabetes prevalence using HbA1c has not been determined. Furthermore, we are aware of no population-based studies examining differences in the prevalence of prediabetes and diabetes according to mutually exclusive categories of ID alone, anemia alone or IDA versus normal iron/hemoglobin. We addressed this gap, finding that fewer women with ID alone versus normal iron/hemoglobin were predicted to have diabetes_HbA1c_ relative to diabetes_FBG_, diabetes_HbA1c+FBG_ or as nondiabetic. Compared with men with normal iron/hemoglobin, however, more men with anemia alone were predicted to have prediabetes_HbA1c_ relative to prediabetes_FBG_, prediabetes_HbA1c+FBG_ or normoglycemia. The direction of association between IDA and diabetes/prediabetes using HbA1c versus FBG were mixed across our statistical models. These findings have important implications for diabetes prevalence estimates across developing countries with a high prevalence of both ID and anemia,^4^ as well as for developed countries, where HbA1c is often considered preferable to FBG for glycemia assessment.^1^

Multiple small, clinic-based studies have examined IDA–HbA1c relationship, however, inconclusive findings from these studies may be because ID and anemia represent two different disease processes. ID, when mild, does not have clinically relevant effects on hemoglobin levels, whereas severe ID results in concurrent ID and anemia.^5^ Though most anemia is due to ID, a large proportion of anemia cases are due to sickle cell disease or other thalassemias, vitamin B12 deficiency or folate deficiency.^5^ Thus, combining ID and anemia in research studies may mask heterogeneous disease processes underlying the relationship between IDA and HbA1c. For example, ID may artificially increase HbA1c by inducing changes to the shape of the hemoglobin molecule, promoting glycation of the terminal valine^8^ or by lowering erythrocyte turnover, allowing more time for glycation of hemoglobin to occur.^6, 7^ Some anemias, on the other hand, may artificially lower HbA1c because of increased erythrocyte turnover.^20^ We know of no population-based studies to date that have addressed these potentially different mechanisms by using two separate measures of glycemia status and mutually exclusive categories of ID alone, anemia alone, IDA or normal iron/hemoglobin.

We found that in our sample, the prevalence of anemia was 7.8% for men and 18.0% for women, which is comparable to national representative data from China.^21^ This prevalence is similar to the prevalence of anemia in Mexico and is much lower than the prevalence for India (~50% for women of reproductive age), but is much higher than the prevalence of anemia in the United States (~7% for women of reproductive age).^4^ Previous studies in China suggest that ID in China may be endemic due to the fact that the majority of the iron consumed in the Chinese diet is from plants, a source of non-heme iron of lower biologic availability compared with animal-source foods.^22^ The higher observed prevalence of ID in women versus men suggests that menstrual blood loss and pregnancy history may be a large contributor to ID in this population. We also found that ~50% of individuals with anemia had concurrent ID, similar to proportions observed in many populations.^5^ It is possible that the other 50% of individuals with anemia could result from genetic hemoglobinopathies, thalassemias or other vitamin deficiencies.

From our multivariable model results, anemia alone and IDA were associated with greater relative risk of prediabetes_HbA1c_ versus normoglycemia in men but not women. A previous population-based study from the US found that among individuals without diabetes, those with anemia versus normal iron/hemoglobin had higher mean HbA1c.^23^ Another found a nonstatistically significant increase in mean HbA1c among individuals with anemia.^9^ However, studies using clinic-based samples from developing countries have shown lower mean HbA1c in individuals with IDA^11^ or anemia^12^ versus normal iron/hemoglobin. These study findings may differ because they were based in regional, small or clinic-based samples. Sex-based differences in our study may be due to the lower prevalence of anemia alone than ID alone in women.

In our sample, we observed that women with ID alone had lower relative risk of being classified with diabetes_HbA1c_ versus as nondiabetic. Similar, although nonsignificant, relationships were seen in men, perhaps owing to sex-based differences in the prevalence and severity of ID and anemia. Our finding confirms results from a clinic-based sample of Indian women, which found lower HbA1c in women with ID versus normal iron,^11^ but contradict findings from a population-based study of US adults, which observed a positive association between ID and HbA1c.^9^

With the exception of our model predicting prediabetes in women, the relationship of IDA (versus normal iron/hemoglobin) with diabetes_HbA1c_ or prediabetes_HbA1c_ (versus normoglycemia), though not statistically significant, was in the same direction as the associations we observed between anemia alone and a higher relative risk of diabetes/prediabetes. It is possible that a positive relationship between IDA (which is severe ID with anemia) and HbA1c is attenuated because ID is negatively associated with HbA1c, while anemia is positively associated with HbA1c. Another possibility is that the low prevalence of diabetes and IDA, particularly in men, could have resulted in insufficient statistical power, or that hemoglobinopathies, thalassemias or other confounding factors could have reduced the precision of our estimates. However, our findings suggest that a different direction of association between ID and HbA1c (negative) versus that between anemia and HbA1c (positive) may underlie inconsistent findings in the epidemiologic literature and support the need to examine both iron and anemia status when assessing diabetes using HbA1c.

Our study contributes to the literature on influences of nonglycemic factors on HbA1c, but there are some limitations of note. In our population-based sample, we do not have an oral glucose tolerance test, the gold standard of glycemic measurement^1^ yet we do have HbA1c and FGB, whereas most studies only collect one or the other. As a nonclinical study, we only have one FBG and HbA1c measurement from participants, which is standard for research studies in the field,^24^ whereas a clinical diagnostic test would include replicate measures. We do not have information on genetic hemoglobinopathies such as HbE, which affects ~30% of Southeast Asians and may artificially lower HbA1c.^18^ However, in sensitivity analysis, we excluded participants from the South region of China where the HbE variant is most prevalent, and observed similar findings in the full sample, although estimates were less precise due to the smaller sample size. Our analysis does not address pregnancy, a time when women are at high risk of developing ID and gestational diabetes. However, our study has the great strengths of reporting population-level associations between ID alone, anemia alone and IDA and diabetes/prediabetes classification in a population-based sample, using a standardized and calibrated technique for HbA1c measurement, allowing us to compare HbA1c across a large and geographically diverse sample; discordant samples and techniques would make this impossible.

Using these unique data, we report the impact of anemia alone, ID or IDA on diabetes and prediabetes prevalence estimates in a sex-stratified, population-based sample of Chinese adults with substantial variability in iron, hemoglobin and diabetes measures. Our findings suggest that ID may result in under-diagnosis of diabetes and anemia may result in over-diagnosis of diabetes when using HbA1c. Furthermore, differences in diabetes/prediabetes classification for individuals with IDA may be driven by the positive association between anemia and HbA1c, but attenuated by the opposing, negative association between ID and HbA1c. Our findings suggest that inconsistent findings in the literature could be due to the complex disease etiology of IDA, and confirm warnings that HbA1c should only be used for glycemic assessment in the absence of ID and/or anemia. These findings suggest that concurrent measurement of iron, hemoglobin and HbA1c is critical to correctly interpret glycaemia status in populations with prevalent ID and/or anemia.

## Figures and Tables

**Table 1:** Percent or mean values for sample characteristics according to sex in Chinese adults aged 18–75 years, 2009 China Health and Nutrition Survey

	*Men* N=*3405*		*Women* N=*3903*		P*-value*^a^
	*Mean or %*	*Standard error*	*Mean or %*	*Standard error*	
FBG mg dl^−1^, mean (s.e.)	97.1	0.5	95	0.4	<0.001
HbA1c %, mean (s.e.)	5.6	0	5.6	0	0.08
Diabetes status,^b^ % (s.e.)					<0.001
Nondiabetic	88.9%	0.5%	90.8%	0.5%	
Diabetes_HbA1c_	2.5%	0.3%	3.0%	0.3%	
Diabetes_FBG_	2.4%	0.3%	1.4%	0.2%	
Diabetes_HbA1c+FBG_	6.2%	0.4%	4.8%	0.3%	
Prediabetes/diabetes status,^c^ % (s.e.)					0.58
Normoglycemia	64.5%	0.9%	65.5%	0.8%	
Prediabetes/diabetes_HbA1c_	15.9%	0.7%	16.2%	0.6%	
Prediabetes/diabetes_FBG_	12.0%	0.6%	11.0%	0.5%	
Prediabetes/diabetes_HbA1c+FBG_	7.6%	0.5%	7.3%	0.4%	
Age, mean (s.e.)	49.0	0.2	49.3	0.2	0.61
Iron/anemia status,^d^ % (s.e.)					<0.001
Normal iron and hemoglobin	79.4%	0.7%	60.1%	0.8%	
Anemia alone	4.6%	0.4%	8.2%	0.4%	
Iron deficiency alone	12.7%	0.6%	22.0%	0.7%	
Iron-deficiency anemia	3.2%	0.3%	9.8%	0.5%	
Waist circumference cm, mean (s.e.)	84.5	0.2	81.1	0.2	<0.001
Urbanization, % (s.e.)^e^					0.43
Low	34.4%	0.8%	33.1%	0.8%	
Medium	33.7%	0.8%	33.9%	0.8%	
High	31.9%	0.8%	33.1%	0.8%	
Income, % (s.e.)					0.06
Low	31.3%	0.8%	33.8%	0.8%	
Medium	34.5%	0.8%	33.6%	0.8%	
High	34.2%	0.8%	32.6%	0.8%	
Region, % (s.e.)					0.85
North	21.1%	0.7%	20.6%	0.6%	
Central	35.6%	0.8%	36.0%	0.8%	
South	43.4%	0.8%	43.4%	0.8%	

Abbreviations: FBG, fasting blood glucose; HbA1c, hemoglobin A1c; s.e., standard error.

a *P*-value for *χ*2 (categorical) or analysis of variance (continuous) test for difference in sample characteristics by sex.

b Diabetes was classified as nondiabetic (FBG <126 mg dl^−1^ and HbA1c <6.5% (48 mmol mol^−1^)), diabetes_HbA1c_ (HbA1c ⩾6.5% (48 mmol mol^−1^) and FBG <126 mg dl^−1^), diabetes_FBG_ (FBG ⩾126 mg dl^−1^ and HbA1c <6.5% (48 mmol mol^−1^)) or diabetes_HbA1c+FBG_ (FBG ⩾126 mg dl^−1^ and HbA1c ⩾6.5% (48 mmol mol^−1^), doctor diagnosis or reported diabetes medication use).

c Prediabetes/diabetes classified as normal (FBG <100 mg dl^−1^ and HbA1c <5.7% (39 mmol mol^−1^)), prediabetes/diabetes_HbA1c_ (HbA1c ⩾5.7% (39 mmol mol^−1^) and FBG <100 mg dl^−1^), prediabetes/diabetes_FBG_ (FBG ⩾100 mg dl^−1^ and HbA1c <5.7% (39 mmol mol^−1^)), or prediabetes/diabetes_HbA1c+FBG_ (FBG ⩾100 mg dl^−1^ and HbA1c ⩾5.7% (39 mmol mol^−1^), doctor diagnosis or reported diabetes medication use).

d The four categories of iron and/or anemia status are defined as follows: normal iron and hemoglobin (hemoglobin ⩾12 g dl^−1^ (women) or ⩾13 g dl^−1^ (men), ferritin ⩾15 μg l^−1^, soluble transferrin receptor <1.76 mg l^−1^), anemia alone (hemoglobin <12 g dl^−1^ (women) or <13 g dl^−1^ (men) without iron deficiency), iron deficiency alone (ferritin <15 μg l^−1^ or transferrin receptor ⩾1.76 mg l^−1^ without anemia) and iron-deficiency anemia (anemia: hemoglobin <12 g dl^−1^ (women) or <13 g dl^−1^ (men) as well as iron-deficiency: ferritin <15 μg l^−1^ or transferrin receptor ⩾1.76 mg l^−1^).

e Column percents may not add to 100% due to rounding.

**Table 2:** Proportion of the sample classified with diabetes and prediabetes by HbA1c only, FBG only, both HbA1c and FBG, or neither according to categories of iron deficiency and/or anemia status, Chinese adults aged 18–75 years, 2009 China Health and Nutrition Survey

*Diabetes classification*	*Nondiabetic*^a^		*Diabetes*_*HbA1c*_^a^		*Diabetes*_*FBG*_^a^		*Diabetes*_*HbA1c+FBG*_^a^		P*-value^b^*
	*Percent*	*Standard* *error*	*Percent*	*Standard error*	*Percent*	*Standard error*	*Percent*	*Standard* *error*	
Men	*N*=3027		*N*=86		*N*=81		*N*=211		0.04
Normal iron/hemoglobin^c^	80.1%	0.7%	81.4%	4.2%	75.3%	4.8%	70.1%	3.2%	
Anemia alone^c^	4.5%	0.4%	5.8%	2.5%	4.9%	2.4%	5.7%	1.6%	
Iron deficiency alone^c^	12.3%	0.6%	9.3%	3.2%	18.5%	4.3%	18.5%	2.7%	
Iron-deficiency anemia^c^	3.1%	0.3%	3.5%	2%	1.2%	1.2%	5.7%	1.6%	
					
Women	*N*=3542		*N*=117		*N*=53		*N*=189		0.008
Normal iron/hemoglobin^c^	59.7%	0.8%	75.2%	4%	54.7%	6.9%	59.8%	3.6%	
Anemia alone^c^	7.9%	0.5%	6.8%	2.3%	11.3%	4.4%	12.7%	2.4%	
Iron deficiency alone^c^	22.3%	0.7%	11.1%	2.9%	26.4%	6.1%	21.7%	3%	
Iron-deficiency anemia^c^	10.1%	0.5%	6.8%	2.3%	7.5%	3.7%	5.8%	1.7%	

*Prediabetes classification*	*Normoglycemia*^a^		*Prediabetes*_*HbA1c*_^a^		*Prediabetes*_*FBG*_^a^		*Prediabetes*_*HbA1c+FBG*_^a^		P-v*alue*^b^
	*Percent*	*Standard* *error*	*Percent*	*Standard error*	*Percent*	*Standard* *error*	*Percent*	*Standard* *error*	
Men	*N*=1952		*N*=480		*N*=364		*N*=231		<0.001
Normal iron/hemoglobin^c^	78.8%	0.9%	78.8%	1.9%	82.7%	2%	90.5%	1.9%	
Anemia alone^c^	4.1%	0.4%	7.7%	1.2%	2.7%	0.9%	3.9%	1.3%	
Iron deficiency alone^c^	13.7%	0.8%	10.0%	1.4%	12.4%	1.7%	4.8%	1.4%	
Iron-deficiency anemia^c^	3.4%	0.4%	3.5%	0.8%	2.2%	0.8%	0.9%	0.6%	
					
Women	*N*=2320		*N*=572		*N*=391		*N*=259		<0.001
Normal iron/hemoglobin^c^	57.6%	1%	66.6%	2%	58.1%	2.5%	65.3%	3%	
Anemia alone^c^	7.9%	0.6%	8.0%	1.1%	9.0%	1.4%	5.8%	1.5%	
Iron deficiency alone^c^	23.5%	0.9%	15.2%	1.5%	25.1%	2.2%	22.8%	2.6%	
Iron-deficiency anemia^c^	10.9%	0.6%	10.1%	1.3%	7.9%	1.4%	6.2%	1.5%	

Abbreviations: FBG, fasting blood glucose; HbA1c, hemoglobin A1c.

a Diabetes was classified as nondiabetic (FBG <126 mg dl^−1^ and HbA1c <6.5% (48 mmol mol^−1^)), diabetes_HbA1c_ (HbA1c ⩾6.5% (48 mmol mol^−1^) and FBG <126 mg dl^−1^), diabetes_FBG_ (FBG ⩾126 mg dl^−1^ and HbA1c <6.5% (48 mmol mol^−1^)) or diabetes_HbA1c+FBG_ (FBG ⩾126 mg dl^−1^ and HbA1c ⩾6.5% (48 mmol mol^−1^), doctor diagnosis or reported diabetes medication use). Prediabetes classification excluded *N*=737 individuals with diabetes according to FBG, HbA1c, doctor diagnosis or reported diabetes medication use. Prediabetes was classified as normoglylcemia (FBG <100 mg dl^−1^ and HbA1c <5.7% (39 mmol mol^−1^)), prediabetes_HbA1c_ (HbA1c=5.7–6.5% (39–48 mmol mol^−1^) and FBG <100 mg dl^−1^), prediabetes_FBG_ (FBG=100–126 mg dl^−1^ and HbA1c <5.7% (39 mmol mol^−1^)) or prediabetes_HbA1c+FBG_ (FBG =100–126 mg dl^−1^ and HbA1c=5.7–6.5% (39–48 mmol mol^−1^)). Column percents may not add up to 100% due to rounding.

b *P*-value for *χ*2 test for differences diabetes or prediabetes classification according to categories of iron deficiency and/or anemia.

c Iron/anemia status was categorized as normal iron and hemoglobin (hemoglobin ⩾12 g dl^−1^ (women) or ⩾13 g dl^−1^ (men), ferritin ⩾15 μg l^−1^, soluble transferrin receptor <1.76 mg l^−1^), anemia alone (as hemoglobin <12 g dl^−1^ (women) or <13 g dl^−1^ (men) without iron deficiency), iron deficiency alone (ferritin <15 μg l^−1^ or transferrin receptor ⩾1.76 mg l^−1^ without anemia) and iron-deficiency anemia (anemia: hemoglobin <12 g dl^−1^ (women) or <13 g dl^−1^ (men), as well as iron-deficiency ferritin <15 μg l^−1^ or transferrin receptor ⩾1.76  mg l^−1^).
