# Supplementary material for: Implications of iron deficiency/anemia on the classification of diabetes using HbA1c
Source: Nutr Diabetes. 2015 Jun 22;5(6):e166–. doi: 10.1038/nutd.2015.16 (PMC4491857; doi:10.1038/nutd.2015.16)

**Supplementary Information**

Implications of iron deficiency/anemia on the classification of diabetes using HbA1c

Running Title: Iron deficiency and/or Anemia and HbA1c

Samantha M Attard, BE,^a^ Amy H Herring, ScD^b,c^, Huijun Wang, MD^d^, Annie-Green Howard, PhD,^b,c^ Amanda L. Thompson, PhD,^b,e^  Linda S Adair, PhD,^a,b^ Elizabeth J Mayer-Davis, PhD,^a,f^ Penny Gordon-Larsen, PhD^a,b^

**Summary:** Supplementary Tables 1 and 2 contain relative risk ratios and confidence intervals from sex-stratified, multinomial logistic models predicting diabetes or prediabetes classification according to HbA1c only, fasting blood glucose (FBG) only, both FBG and HbA1c, or neither FBG nor HbA1c. Supplementary Figure 1 contains the distribution of hemoglobin in the sample for men and women.

**Supplementary Table 1. Relative risk ratios (95% Confidence Interval) from sex-stratified multinomial logistic models predicting diabetes classification in Chinese adults from the 2009 China Health and Nutrition Survey^*^**

|  | **Men** | | | **Women** | | |
| --- | --- | --- | --- | --- | --- | --- |
|  | Anemia alone^†^ | **Iron deficiency alone^†^** | **Iron deficiency anemia^†^** | **Anemia alone^†^** | **Iron deficiency alone^†^** | **Iron deficiency anemia^†^** |
| **Nondiabtic Referent**^‡^ | |  |  |  |  |  |
| Nondiabetic | 1.00 | 1.00 | 1.00 | 1.00 | 1.00 | 1.00 |
| HbA1c Only | 1.24 (0.48, 3.20) | 0.85 (0.40, 1.80) | 1.27 (0.38, 4.19) | 0.62 (0.29, 1.32) | 0.52 (0.29, 0.95) | 0.96 (0.45, 2.06) |
| FBG Only | 0.98 (0.35, 2.78) | 1.60 (0.89, 2.88) | 0.41 (0.06, 3.00) | 1.38 (0.55, 3.43) | 1.42 (0.74, 2.72) | 1.08 (0.37, 3.15) |
| HbA1c+FBG | 1.41 (0.74, 2.69) | 1.80 (1.21, 2.67) | 2.38 (1.20, 4.72) | 1.55 (0.95, 2.55) | 1.22 (0.83, 1.81) | 1.22 (0.63, 2.37) |
| **HbA1c Only Referent**^‡^ | |  |  |  |  |  |
| Nondiabetic | 0.8 (0.31, 2.07) | 1.18 (0.56, 2.50) | 0.79 (0.24, 2.62) | 1.62 (0.76, 3.46) | 1.92 (1.05, 3.50) | 1.04 (0.49, 2.22) |
| HbA1c Only | 1.00 | 1.00 | 1.00 | 1.00 | 1.00 | 1.00 |
| FBG Only | 0.79 (0.20, 3.13) | 1.89 (0.74, 4.81) | 0.32 (0.03, 3.22) | 2.23 (0.70, 7.16) | 2.72 (1.13, 6.52) | 1.12 (0.31, 4.11) |
| HbA1c+FBG | 1.14 (0.38, 3.42) | 2.12 (0.93, 4.84) | 1.88 (0.50, 7.00) | 2.52 (1.06f) | 2.35 (1.17, 4.71) | 1.27 (0.48, 3.37) |
| **FBG Only Referent**^‡^ | |  |  |  |  |  |
| Nondiabetic | 1.02 (0.36, 2.90) | 0.62 (0.35, 1.12) | 2.45 (0.33, 18.08) | 0.73 (0.29, 1.81) | 0.71 (0.37, 1.35) | 0.92 (0.32, 2.69) |
| HbA1c Only | 1.27 (0.32, 5.05) | 0.53 (0.21, 1.35) | 3.1 (0.31, 31.06) | 0.45 (0.14, 1.44) | 0.37 (0.15, 0.88) | 0.89 (0.24, 3.25) |
| FBG Only | 1.00 | 1.00 | 1.00 | 1.00 | 1.00 | 1.00 |
| HbA1c+FBG | 1.44 (0.44, 4.76) | 1.12 (0.57, 2.23) | 5.84 (0.73, 46.84) | 1.13 (0.41, 3.10) | 0.86 (0.41, 1.81) | 1.13 (0.33, 3.90) |
| **HbA1c+FBG Referent**^‡^ | |  |  |  |  |  |
| Nondiabetic | 0.71 (0.37, 1.35) | 0.56 (0.37, 0.83) | 0.42 (0.21, 0.83) | 0.64 (0.39, 1.05) | 0.82 (0.55, 1.20) | 0.82 (0.42, 1.58) |
| HbA1c Only | 0.88 (0.29, 2.64) | 0.47 (0.21, 1.07) | 0.53 (0.14, 1.98) | 0.40 (0.17, 0.94) | 0.43 (0.21, 0.85) | 0.79 (0.30, 2.08) |
| FBG Only | 0.69 (0.21, 2.28) | 0.89 (0.45, 1.76) | 0.17 (0.02, 1.38) | 0.89 (0.32, 2.44) | 1.16 (0.55, 2.43) | 0.88 (0.26, 3.04) |
| HbA1c+FBG | 1.00 | 1.00 | 1.00 | 1.00 | 1.00 | 1.00 |

^*^ Multinomial logistic model predicting diabetes classification, with categories of iron deficiency and/or anemia status as the main exposure, adjusting for age, urbanicity level (low, medium, high), number of cigarettes smoked per day and number of cigarettes squared (men only), ever pregnant (ever versus never; women only), household income (low, medium, high), region (north, central, south), and waist circumference and clustered at the community level.

^†^The categories of iron/anemia status are as follows: normal iron/hemoglobin (hemoglobin ≥12 g/dl (women) or ≥13 g/dl (men), ferritin ≥15 μg/l, soluble transferrin receptor <1.76 mg/l), anemia alone (as hemoglobin <12 g/dl (women) or <13 g/dl (men) without iron deficiency), iron deficiency alone (ferritin<15μg/l or transferrin receptor ≥1.76 mg/l without anemia), and iron deficiency anemia (anemia: hemoglobin<12 g/dl (women) or <13 g/dl (men), as well as iron-deficiency: ferritin <15 μg/l or transferrin receptor ≥1.76 mg/l). The referent exposure (not shown) is normal iron/hemoglobin.

^‡^Diabetes outcome was classified as: nondiabetic (FBG<126 mg/dl and HbA1c<6.5% [48 mmol/mol]), HbA1c only (HbA1c≥6.5% [48 mmol/mol] and FBG<126 mg/dl), FBG only (FBG≥126 mg/dl and HbA1c<6.5% [48 mmol/mol]), or HbA1c+FBG (FBG≥126 mg/dl and HbA1c≥6.5% [48 mmol/mol] or doctor diagnosis of diabetes).

**Supplementary Table 2. Odds ratios (95% Confidence Interval) from sex-stratified multinomial logistic models predicting prediabetes classification in Chinese adults from the 2009 China Health and Nutrition Survey^*^**

|  | **Men** | | | **Women** | | |
| --- | --- | --- | --- | --- | --- | --- |
|  | **Anemia alone^†^** | **Iron deficiency alone^†^** | **Iron deficiency anemia^†^** | **Anemia alone^†^** | **Iron deficiency alone^†^** | **Iron deficiency anemia^†^** |
| **Normoglycemia Referent**^‡^ | |  |  |  |  |  |
| Normoglycemia | 1.00 | 1.00 | 1.00 | 1.00 | 1.00 | 1.00 |
| HbA1c Only | 1.81 (1.16, 2.82) | 0.84 (0.59, 1.19) | 1.18 (0.65, 2.12) | 1.00 (0.69, 1.45) | 0.64 (0.49, 0.84) | 1.22 (0.88, 1.70) |
| FBG Only | 0.60 (1.31, 1.19) | 0.84 (0.59, 1.18) | 0.65 (0.31, 1.40) | 1.00 (0.67, 1.50) | 1.13 (0.87, 1.48) | 0.88 (0.58, 1.32) |
| HbA1c+FBG | 0.76 (0.36, 1.59) | 0.33 (0.17, 0.62) | 0.23 (0.05, 0.98) | 0.72 (0.40, 1.29) | 1.05 (0.75, 1.47) | 1.02 (0.58, 1.79) |
| **HbA1c Only Referent**^‡^ | |  |  |  |  |  |
| Normoglycemia | 0.55 (0.35, 0.86) | 1.19 (0.84, 1.69) | 0.85 (0.47, 1.53) | 1.00 (0.69, 1.45) | 1.56 (1.19, 2.03) | 0.82 (0.59, 1.14) |
| HbA1c Only | 1.00 | 1.00 | 1.00 | 1.00 | 1.00 | 1.00 |
| FBG Only | 0.33 (0.16, 0.70) | 0.99 (0.63, 1.56) | 0.56 (0.23, 1.34) | 1.00 (0.61, 1.65) | 1.76 (1.25, 2.49) | 0.72 (0.44, 1.17) |
| HbA1c+FBG | 0.42 (0.20, 0.90) | 0.39 (0.20, 0.77) | 0.20 (0.04, 0.87) | 0.72 (0.39, 1.34) | 1.64 (1.12, 2.40) | 0.82 (0.46, 1.52) |
| **FBG Only Referent**^‡^ | |  |  |  |  |  |
| Normoglycemia | 1.66 (0.84, 3.28) | 1.20 (0.85, 1.70) | 1.53 (0.72, 3.27) | 1.00 (0.67,1.49) | 0.88 (0.68,1.17) | 1.14 (0.76,1.71) |
| HbA1c Only | 3.00 (1.43, 6.30) | 1.01 (0.64, 1.58) | 1.80 (0.74, 4.35) | 1.00 (0.61,1.64) | 0.57 (0.40,0.80) | 1.39 (0.85,2.27) |
| FBG Only | 1.00 | 1.00 | 1.00 | 1.00 | 1.00 | 1.00 |
| HbA1c+FBG | 1.26 (0.49, 3.24) | 0.39 (0.20, 0.79) | 0.35 (0.07, 1.72) | 0.72 (0.37,1.40) | 0.93 (0.63,1.38) | 1.16 (0.60,2.25) |
| **HbA1c+FBG Referent**^‡^ | |  |  |  |  |  |
| Normoglycemia | 1.32 (0.63, 2.75) | 3.05 (1.61, 5.79) | 2.55 (1.27, 5.12) | 1.39 (0.78, 2.48) | 0.95 (0.68, 1.33) | 1.08 (0.73, 1.60) |
| HbA1c Only | 2.38 (1.11, 5.09) | 2.57 (1.30, 5.09) | 5.10 (1.15, 22.59) | 1.39 (0.75, 2.59) | 0.61 (0.42, 0.90) | 1.19 (0.66, 2.17) |
| FBG Only | 0.79 (0.31, 2.03) | 2.55 (1.27, 5.12) | 2.83 (0.58, 13.81) | 1.39 (0.72, 2.70) | 1.08 (0.73, 1.60) | 0.86 (0.44, 1.66) |
| HbA1c+FBG | 1.00 | 1.00 | 1.00 | 1.00 | 1.00 | 1.00 |

^*^Multinomial logistic model predicting prediabetes classification, with categories of iron deficiency and/or anemia status as the main exposure, adjusting for age, urbanicity level (low, medium, high), number of cigarettes smoked per day and number of cigarettes squared (men only), ever pregnant (ever versus never; women only), household income (low, medium, high), region (north, central, south), and waist circumference and clustered at the community level.

^†^The categories of iron/anemia status are as follows: normal iron/hemoglobin (hemoglobin ≥12 g/dl (women) or ≥13 g/dl (men), ferritin ≥15 μg/l, soluble transferrin receptor <1.76 mg/l), anemia alone (as hemoglobin <12 g/dl (women) or <13 g/dl (men) without iron deficiency), iron deficiency alone (ferritin<15μg/l or transferrin receptor ≥1.76 mg/l without anemia), and iron deficiency anemia (anemia: hemoglobin<12 g/dl (women) or <13 g/dl (men), as well as iron-deficiency: ferritin <15 μg/l or transferrin receptor ≥1.76 mg/l). The referent exposure (not shown) is normal iron/hemoglobin.

^‡^Prediabetes model excluded N=737 individuals with diabetes according to FBG, HbA1c, doctor diagnosis, or diabetes medication use. Prediabetes outcome was classified as: normoglycemia (FBG<100 mg/dl and HbA1c<5.7% [39 mmol/mol]), HbA1c only (HbA1c 5.7-6.5% [39-48 mmol/mol] and FBG<100 mg/dl), FBG only (FBG 100-126 mg/dl and HbA1c<5.7% [39 mmol/mol]), or HbA1c+FBG (FBG 100-126 mg/dl and HbA1c≥5.7% [39-48 mmol/mol]

**Supplementary Figure 1. Distribution of hemoglobin for men (left panel) and women (right panel) from the 2009 China Health and Nutrition Survey**


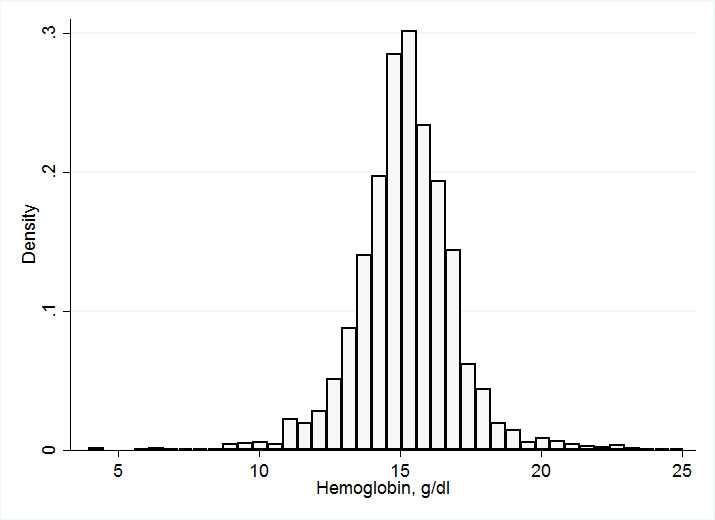

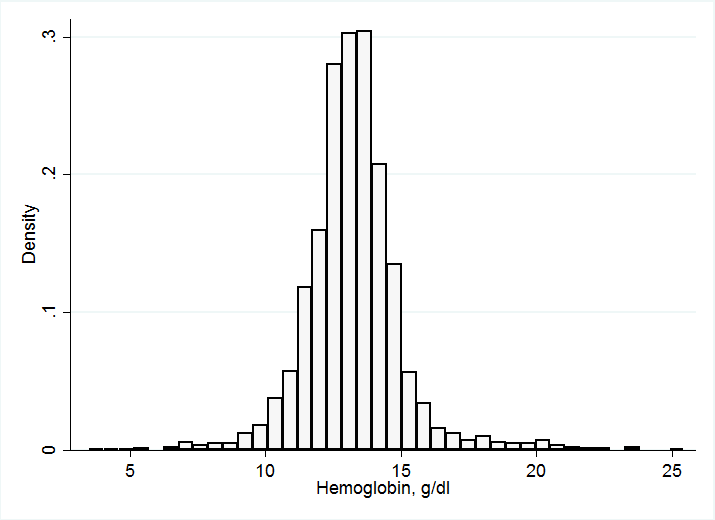

Supplement: Supplementary Information [file nutd201516x1.doc]
